# Red Dot Basal Cell Carcinoma: Report of Cases and Review of This Unique Presentation of Basal Cell Carcinoma

**DOI:** 10.7759/cureus.1110

**Published:** 2017-03-22

**Authors:** Philip R Cohen

**Affiliations:** 1 Department of Dermatology, University of California, San Diego

**Keywords:** basal, cell, cancer, carcinoma, dot, red, skin

## Abstract

Red dot basal cell carcinoma is a unique variant of basal cell carcinoma. Including the three patients described in this report, red dot basal cell carcinoma has only been described in seven individuals. This paper describes the features of two males and one female with red dot basal cell carcinoma and reviews the characteristics of other patients with this clinical subtype of basal cell carcinoma. A 70-year-old male developed a pearly-colored papule with a red dot in the center on his nasal tip. A 71-year-old male developed a red dot surrounded by a flesh-colored papule on his left nostril. Lastly, a 74-year-old female developed a red dot within an area of erythema on her left mid back. Biopsy of the lesions all showed nodular and/or superficial basal cell carcinoma. Correlation of the clinical presentation and pathology established the diagnosis of red dot basal cell carcinoma. The tumors were treated by excision using the Mohs surgical technique. Pubmed was searched with the keyword: basal, cell, cancer, carcinoma, dot, red, and skin. The papers generated by the search and their references were reviewed. Red dot basal cell carcinoma has been described in three females and two males; the gender was not reported in two patients. The tumor was located on the nose (five patients), back (one patient) and thigh (one patient). Cancer presented as a solitary small red macule or papule; often, the carcinoma was surrounded by erythema or a flesh-colored papule. Although basal cell carcinomas usually do not blanch after a glass microscope slide is pressed against them, the red dot basal cell carcinoma blanched after diascopy in two of the patients, resulting in a delay of diagnosis in one of these individuals. Dermoscopy may be a useful non-invasive modality for evaluating skin lesions when the diagnosis of red dot basal cell carcinoma is considered. Mohs surgery is the treatment of choice; in some of the patients, the ratio of the area of the postoperative wound to that of the preoperative cancer was greater than 12:1, demonstrating a significant lateral spread of the tumor beyond the observed clinical margins of the neoplasm. In conclusion, in a patient with a personal history of actinic keratosis or nonmelanoma skin cancer, the appearance of a new red dot in a sun-exposed site should prompt additional evaluation of the skin lesion to exclude or establish the diagnosis of red dot basal cell carcinoma.

## Introduction

Red dot basal cell carcinoma is a unique variant of basal cell carcinoma [1-2]. Its appearance can mimic that of a benign vascular lesion such as a hemangioma or a telangiectasia; hence, if the possibility of a red dot basal cell carcinoma is not suspected, there can be a delay in establishing the diagnosis [3-4]. Three patients with red dot basal cell carcinoma are described herein and the characteristics of this morphologic subtype of basal cell carcinoma, not only in these patients but also in the previously reported individuals with this basal cell carcinoma variant, are summarized. Informed consent statements were obtained for this study.

## Case presentation

### Case 1

A 70-year-old male presented for a complete skin examination in July 2016. He had a history of actinic keratoses (AK) and three basal cell carcinomas. He had new skin lesions on his nose and back.

His past medical history is significant for benign paroxysmal positional vertigo (BPPV), hyperlipidemia (treated with atorvastatin), gout (treated with allopurinol), nephrolithiasis and recent stenting of his right coronary artery one month ago and currently receiving (clop nephrolithiasis dogrel).

**Figure 1:**
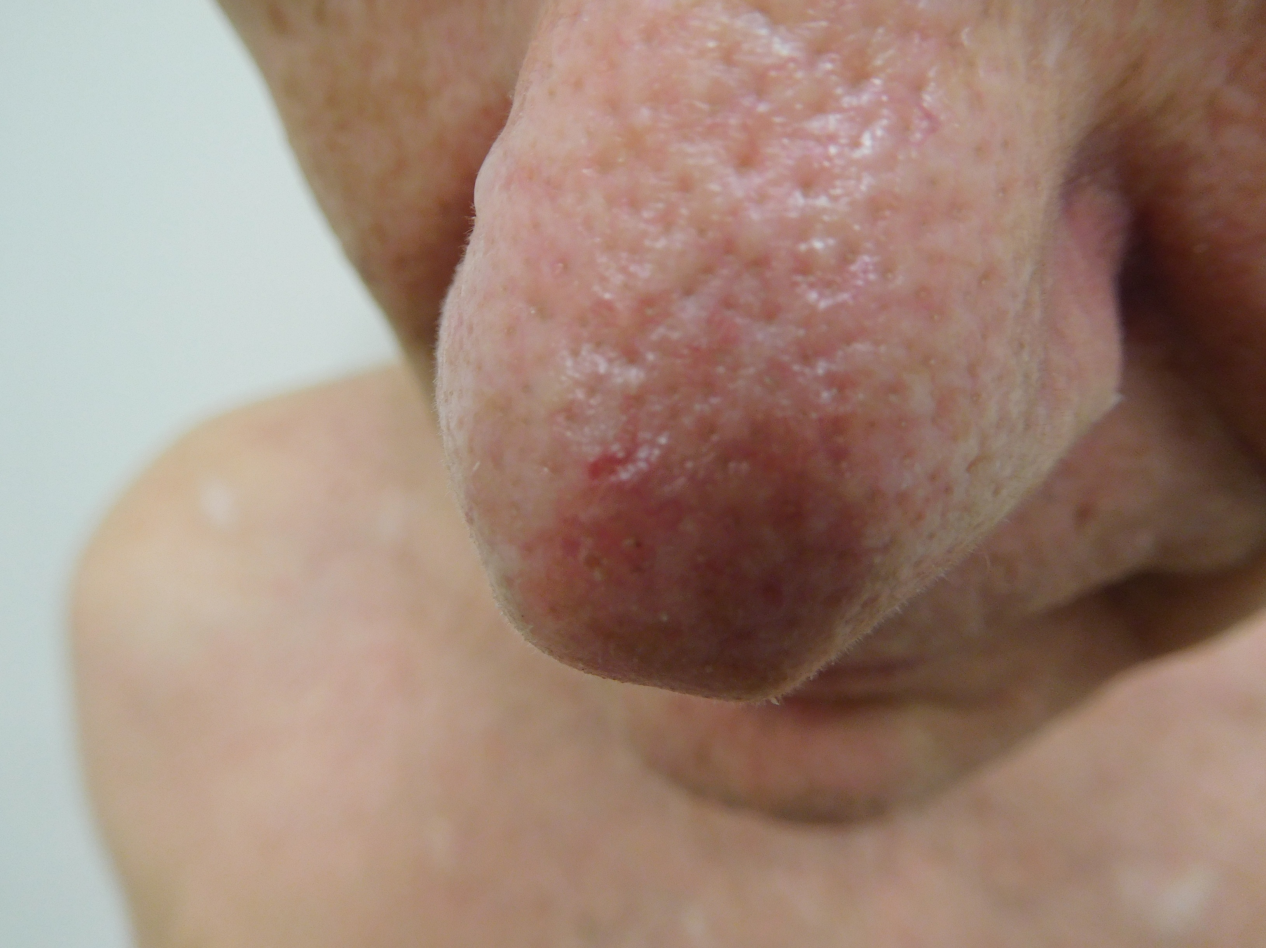
Clinical presentation of a red dot basal cell carcinoma on the nose of a 70-year-old male A red dot basal cell carcinoma presenting as a 1 x 1 mm red dot in the center of a 3 x 3 mm pearly-colored papule on the nasal tip of a 70-year-old male.

**Figure 2:**
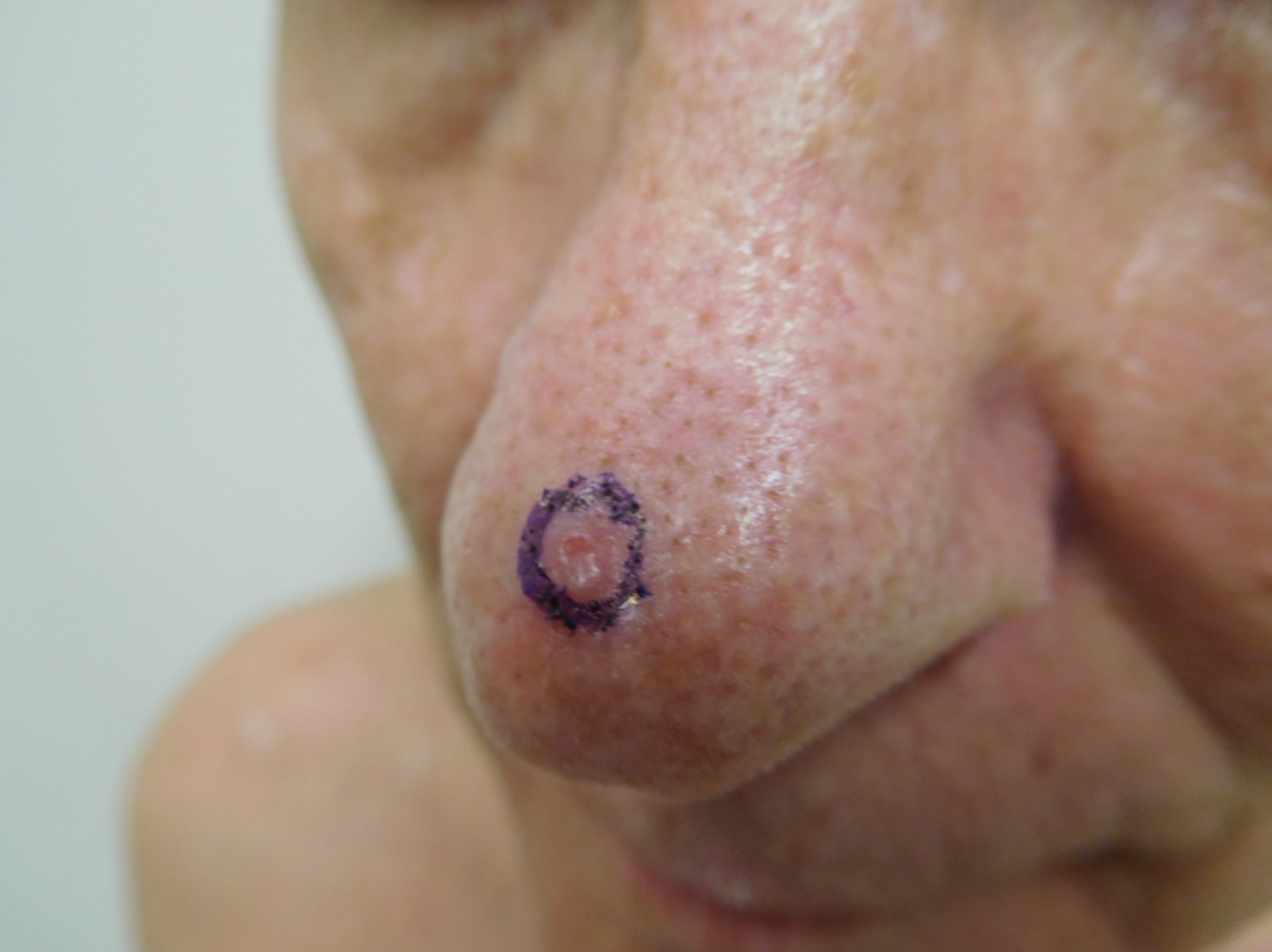
Clinical presentation of a red dot basal cell carcinoma on the nose of a 70-year-old male The pearly-colored papule with the central red dot on his nose -the red dot basal cell carcinoma - is circled to clearly demarcate the clinical margins of the tumor.

**Figure 3:**
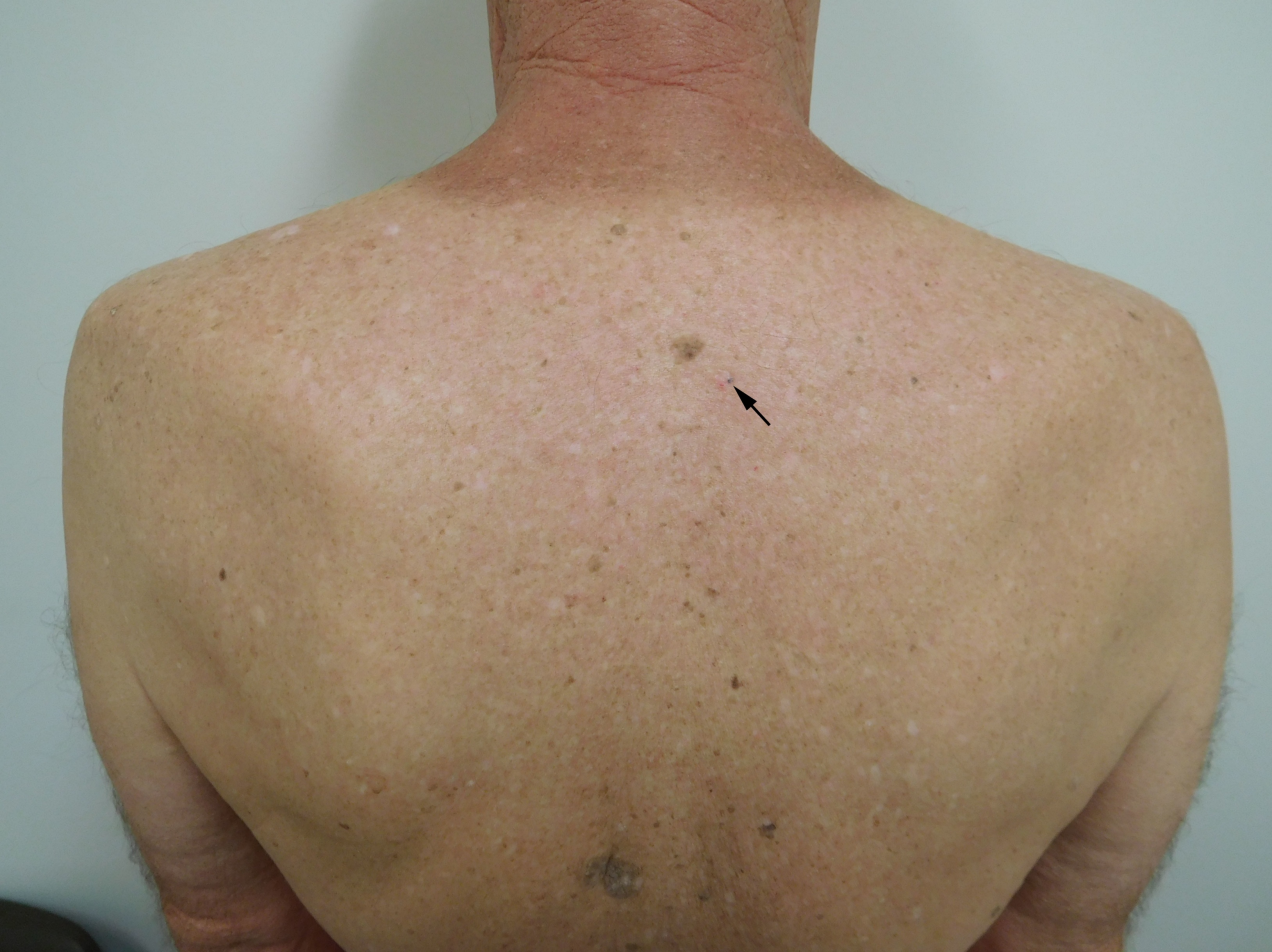
Clinical presentation of a pigmented basal cell carcinoma on the back of a 70-year-old male Distant view of the patient's back shows a pigmented basal cell carcinoma presenting as a 1 x 1 mm black macule (arrow). The pigmented basal cell carcinoma is located on the upper mid back. A prominent brown seborrheic keratosis on the upper mid back is located below the patient's tan line. The pigmented basal cell carcinoma is located to the right and inferior to the brown seborrheic keratosis; it is adjacent to the inferior border of a white plaque and to the right of the red excoriation.

**Figure 4:**
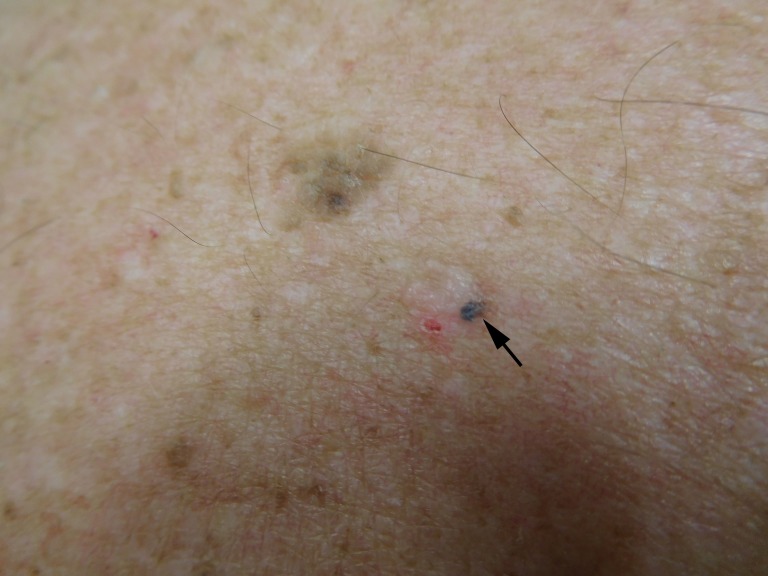
Clinical presentation of a pigmented basal cell carcinoma on the back of a 70-year-old male Closer view of the pigmented basal cell carcinoma on the patient's back appears as a 1 x 1 mm black macule located on the inferior border of the white plaque and to the right of the red excoriation (arrow). A prominent brown seborrheic keratosis is noted superiorly and to the left of the pigmented basal cell carcinoma.

The cutaneous examination was remarkable for 22 erythematous scaling plaques on his scalp, face, and arms, consistent clinically with actinic keratoses. He had yellow longitudinal streaks on his left great and fifth toe, consistent with onychomycosis. On his nasal tip, there was a 3 x 3 mm pearly-colored papule with a red dot of less than 1 mm in the center (Figures 1-2). In addition, there was a 1 x 1 mm black macule with an adjacent 6 x 6 mm white plaque on his upper mid back (Figures 3-4).

**Figure 5:**
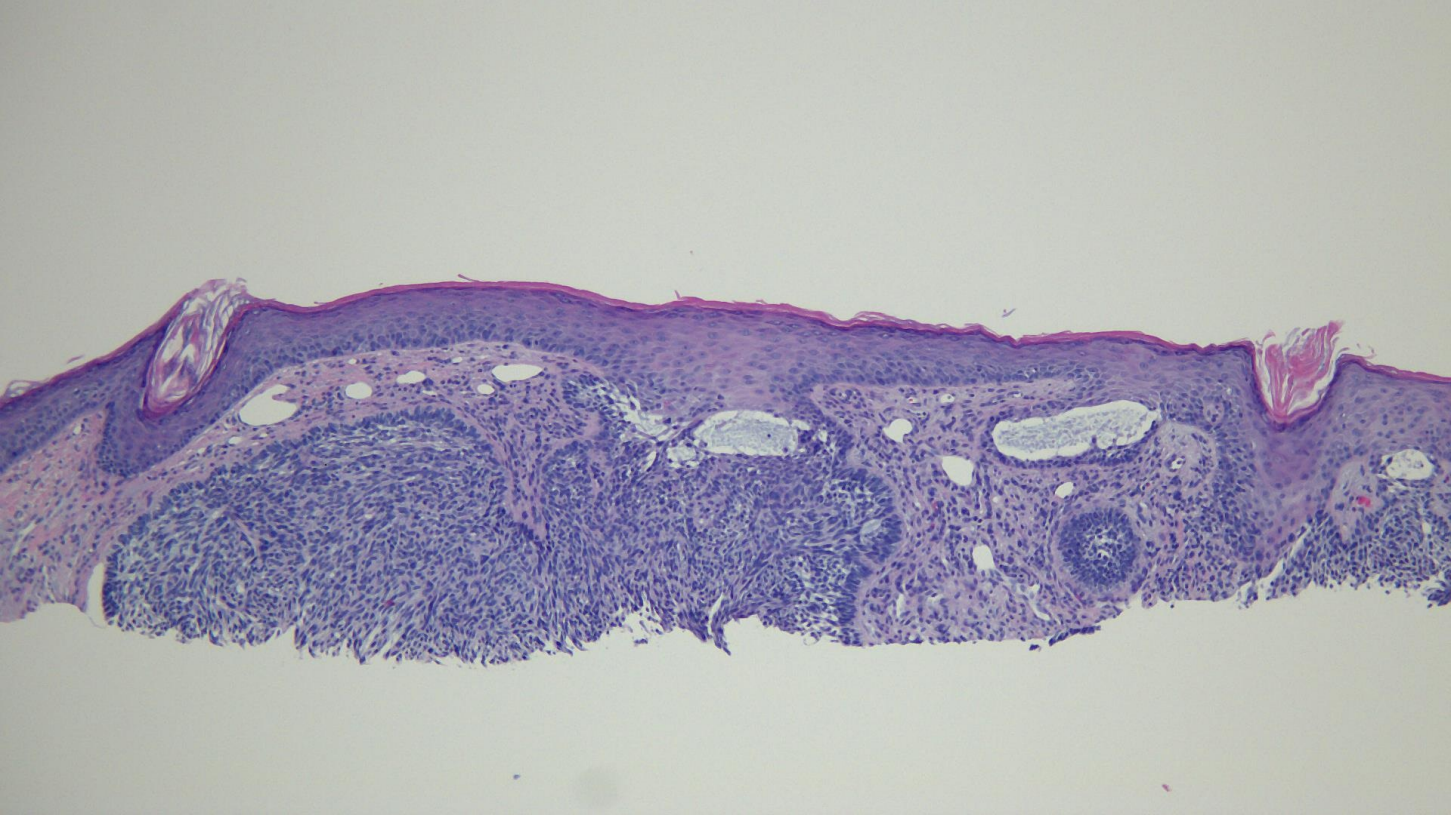
Microscopic examination of a red dot basal cell carcinoma on the nose of a 70-year-old male Low magnification view of the biopsy of the red dot basal cell carcinoma from the tip of the nose shows nodular aggregates and superficial buds of basaloid tumor cells extending from the overlying epidermis into the underlying dermis (hematoxylin and eosin, x 10).

**Figure 6:**
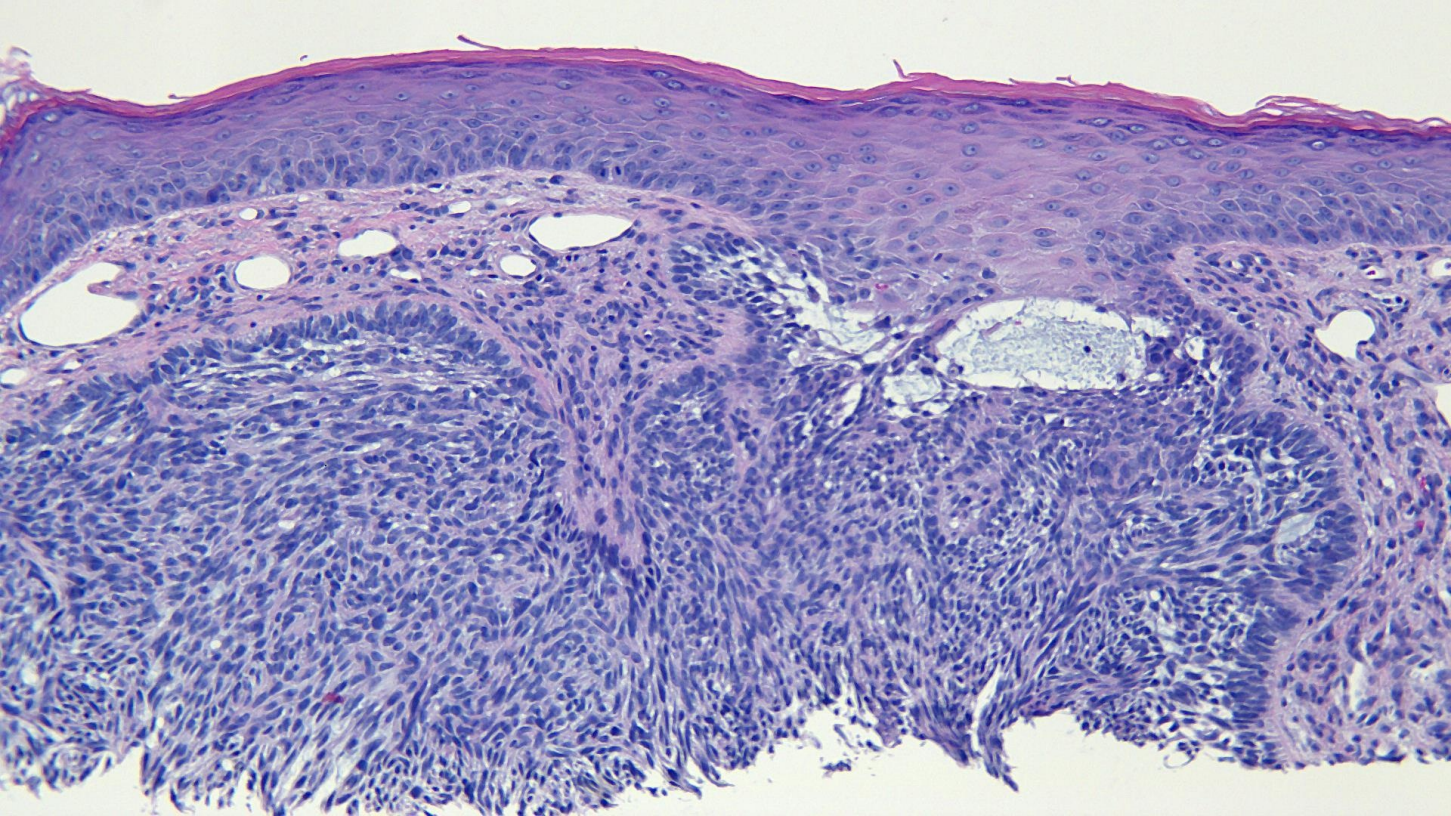
Microscopic examination of a red dot basal cell carcinoma on the nose of a 70-year-old male Intermediate magnification view of the biopsy of the red dot basal cell carcinoma from the tip of the nose shows that the stroma adjacent to the tumor in the papillary dermis contains several telangiectatic blood vessels (hematoxylin and eosin, x 20).

**Figure 7:**
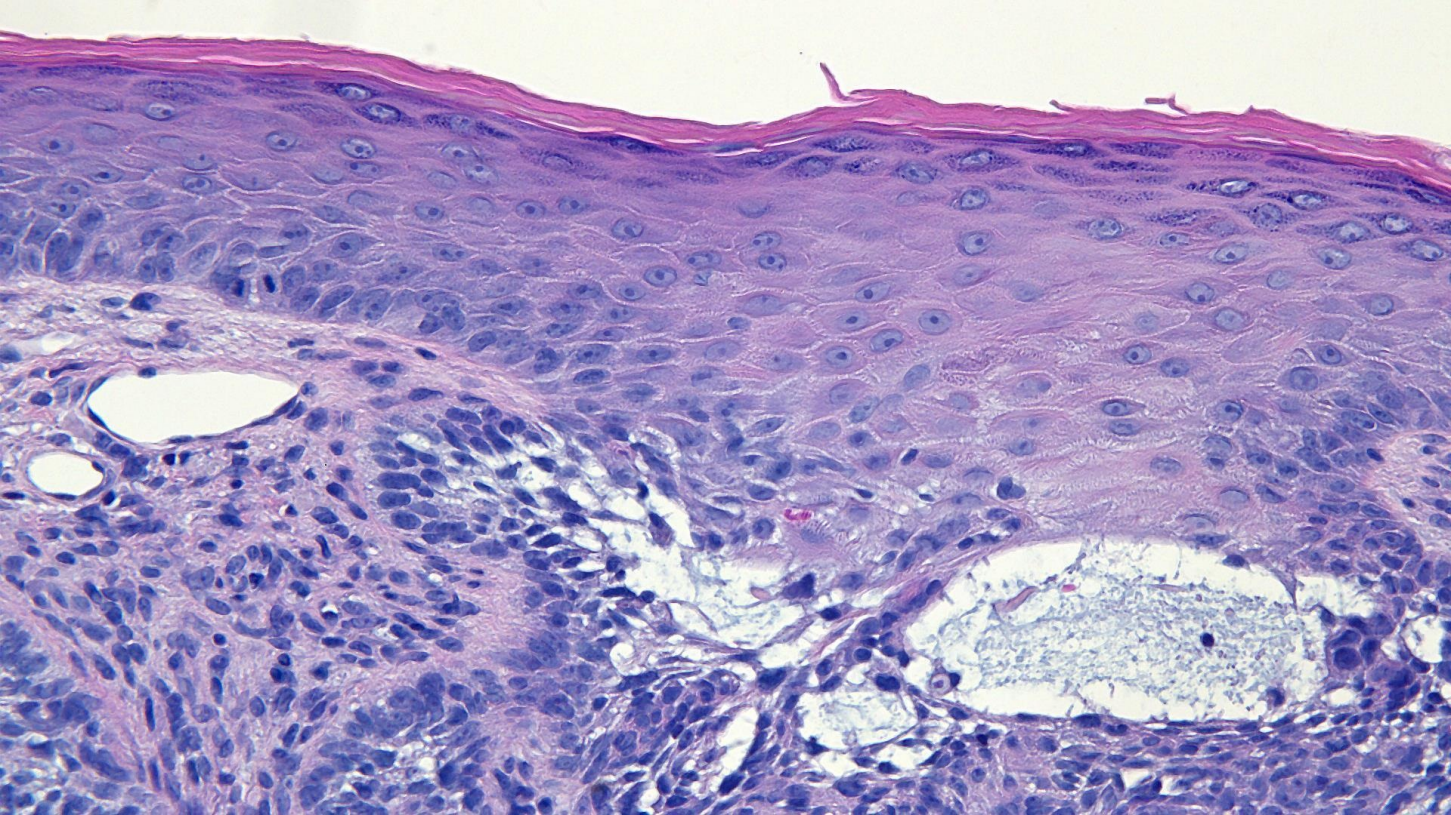
Microscopic examination of a red dot basal cell carcinoma on the nose of a 70-year-old male High magnification view of the biopsy of the red dot basal cell carcinoma from the tip of the nose shows that mucin is present within the tumor (hematoxylin and eosin, x 40).

The actinic keratoses were treated with liquid nitrogen cryotherapy and the patient declined treatment for his toenail fungal infection. The back and nose lesions were each biopsied using a punch and shave technique, respectively. The nasal tip lesion red dot showed superficial buds and nodular aggregates of basaloid tumor cells extending from the epidermis into the dermis; there were also telangiectatic blood vessels in the papillary dermis (Figures 5-7). The upper mid back lesion showed an atypical proliferation of basaloid cells both emanating from the epidermis as oblong clusters and as discrete nests in the dermis; focally, some of the basaloid cells harbored cytoplasmic melanin pigment.

Correlation of the clinical presentation and pathologic findings of the nasal tip lesion was a red dot basal cell carcinoma (with superficial and nodular tumor aggregates). The tumor was excised using the Mohs technique. Two stages were required for cancer removal; the size of the defect was 11 x 10 mm. A full-thickness skin graft was used to repair the surgical wound.

The pigmented superficial and nodular basal cell carcinoma on his upper mid back was excised and the surgical wounds were closed with complex layered closure. Follow-up examination five months later showed excellent healing of both surgical sites and no evidence of recurrence.

### Case 2

A 71-year-old male presented for a complete skin examination in November 2016. He had a history of actinic keratoses, a basal cell carcinoma, and a squamous cell carcinoma. He had new skin lesions on his nose.

His past medical history was significant for hyperlipidemia (treated with simvastatin) and coronary artery disease (and currently receiving clopidogrel).

**Figure 8:**
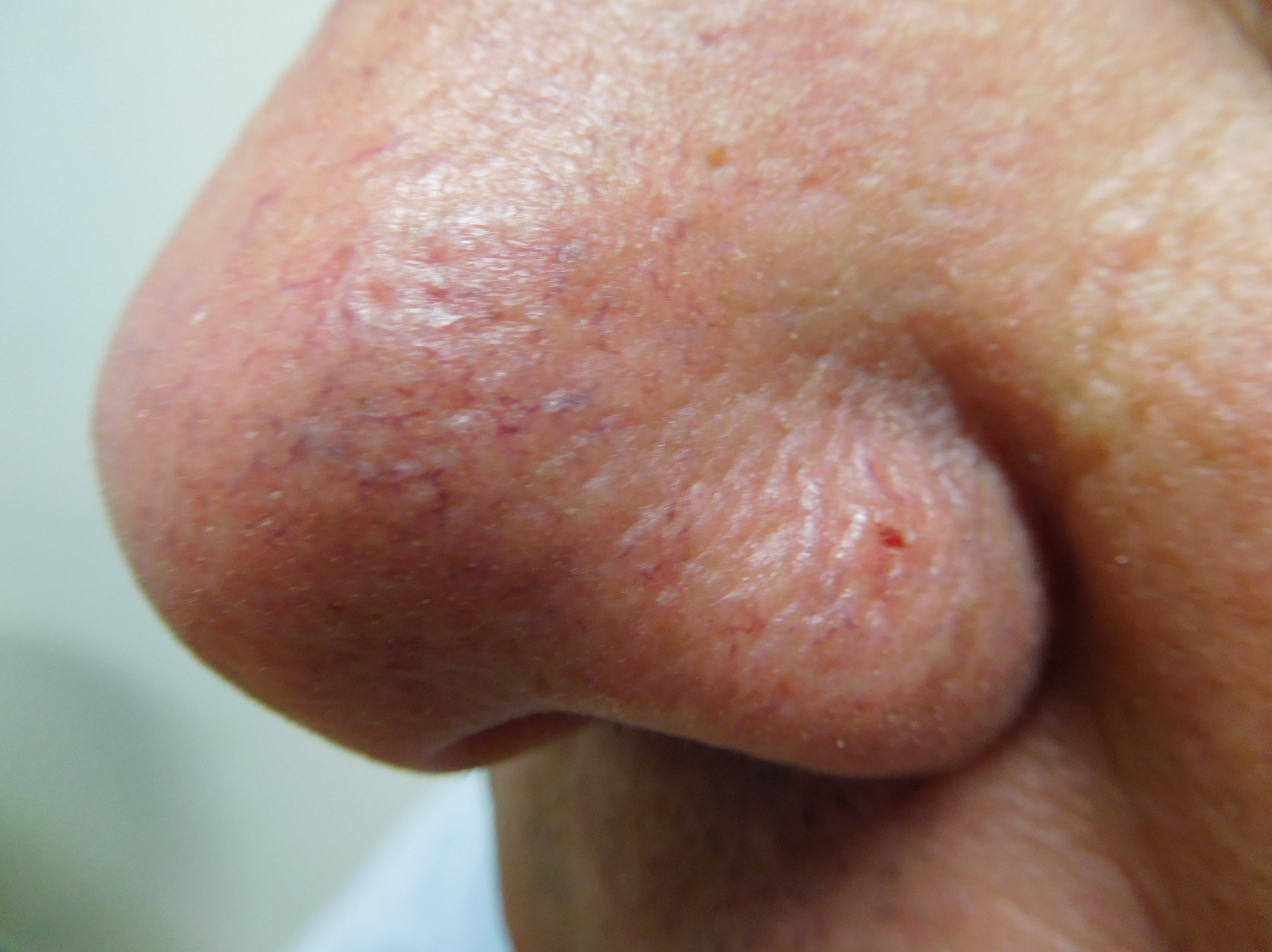
Clinical presentation of a red dot basal cell carcinoma on the left nostril of a 71-year-old male A red dot basal cell carcinoma presenting as a 1 x 1 mm red dot surrounded by a 2 x 2 mm flesh-colored papule on the left nostril of a 71-year-old male.

**Figure 9:**
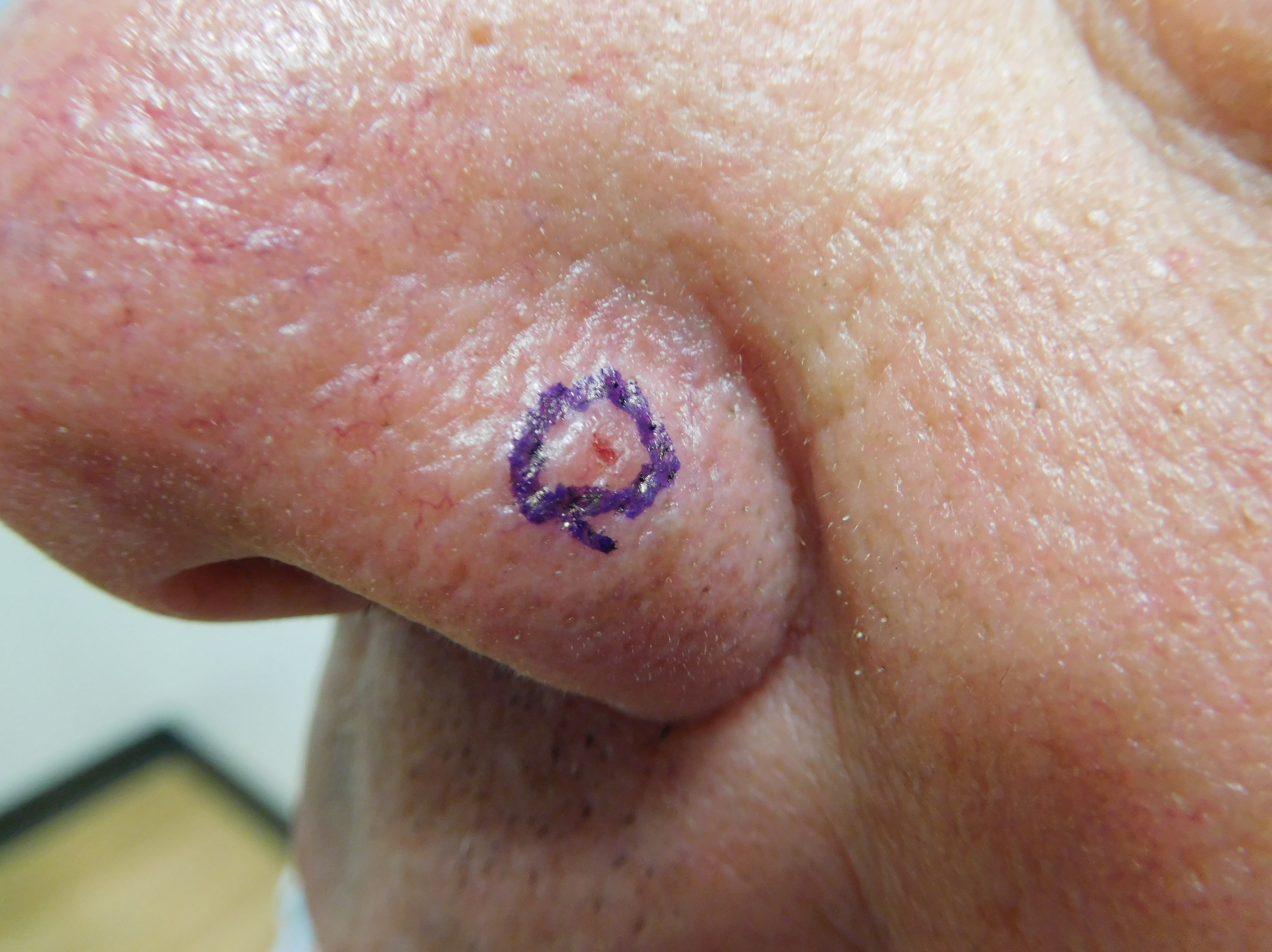
Clinical presentation of a red dot basal cell carcinoma on the left nostril of a 71-year-old male The flesh-colored papule with the central red dot on the nose - the red dot basal cell carcinoma - is circled to clearly demarcate the clinical margins of the tumor.

**Figure 10:**
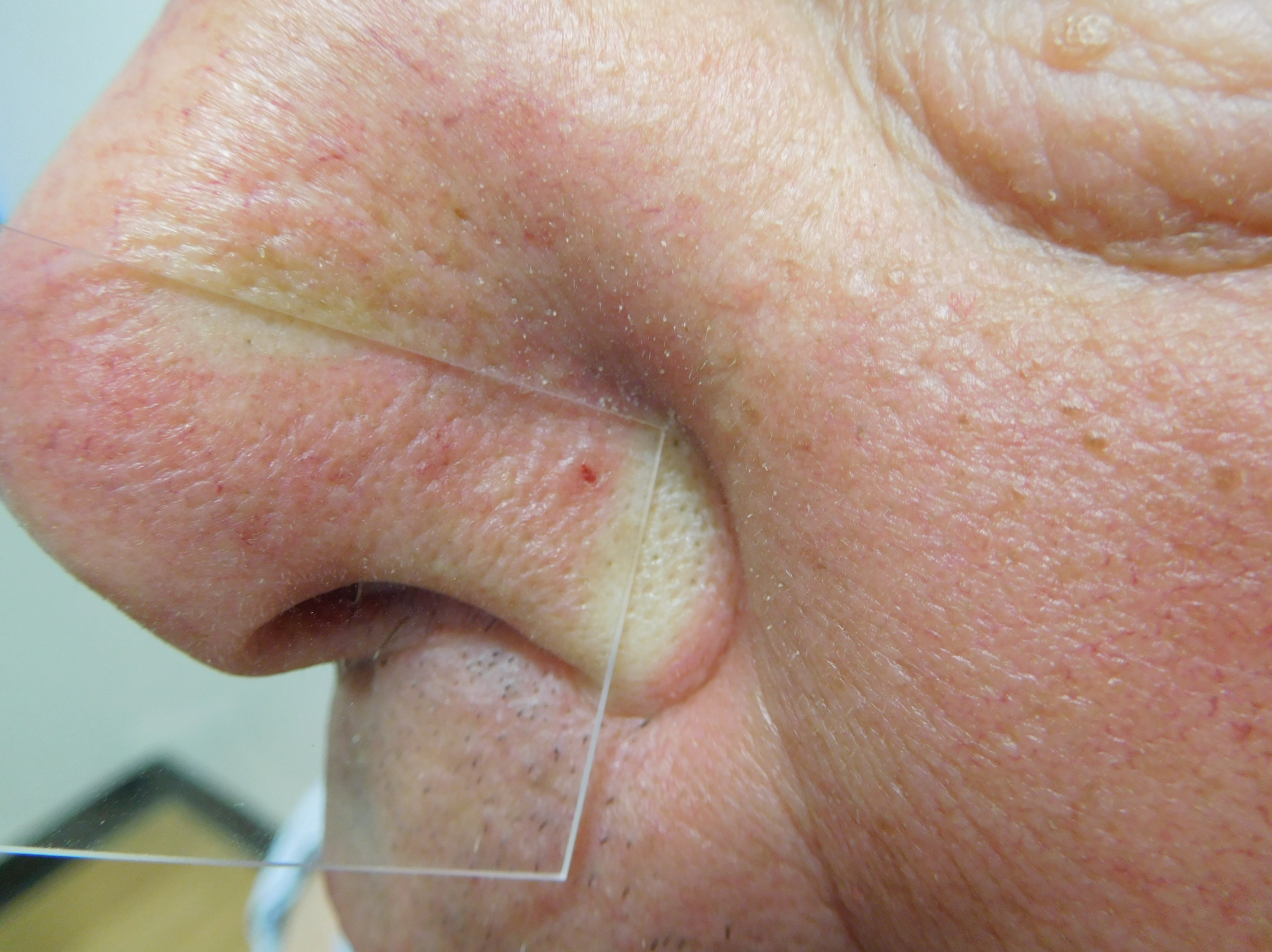
Diascopy of a red dot basal cell carcinoma on the left nostril of a 71-year-old male The left nostril tumor does not blanch when a glass microscope slide is pressed against it.

The cutaneous examination was remarkable for 11 erythematous scaling plaques on his face and arms, consistent clinically with actinic keratoses. There is a 1 x 1 mm red dot surrounded by a 2 x 2 mm flesh-colored papule on his left nostril (Figures 8-9). The lesion did not blanch when pressed with a glass microscope slide (Figure 10).

**Figure 11:**
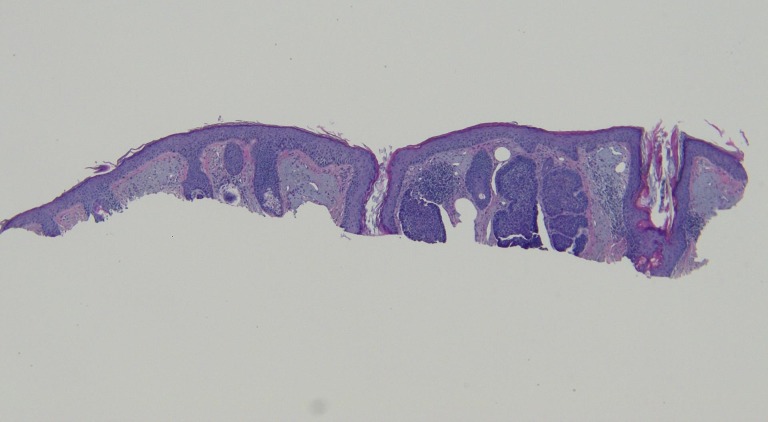
Microscopic examination of a red dot basal cell carcinoma on the left nostril of a 71-year-old male Low magnification view of the biopsy of the red dot basal cell carcinoma from the left nostril shows nodular aggregates of basaloid tumor cells extending from the overlying epidermis into the underlying dermis (hematoxylin and eosin, x 4).

**Figure 12:**
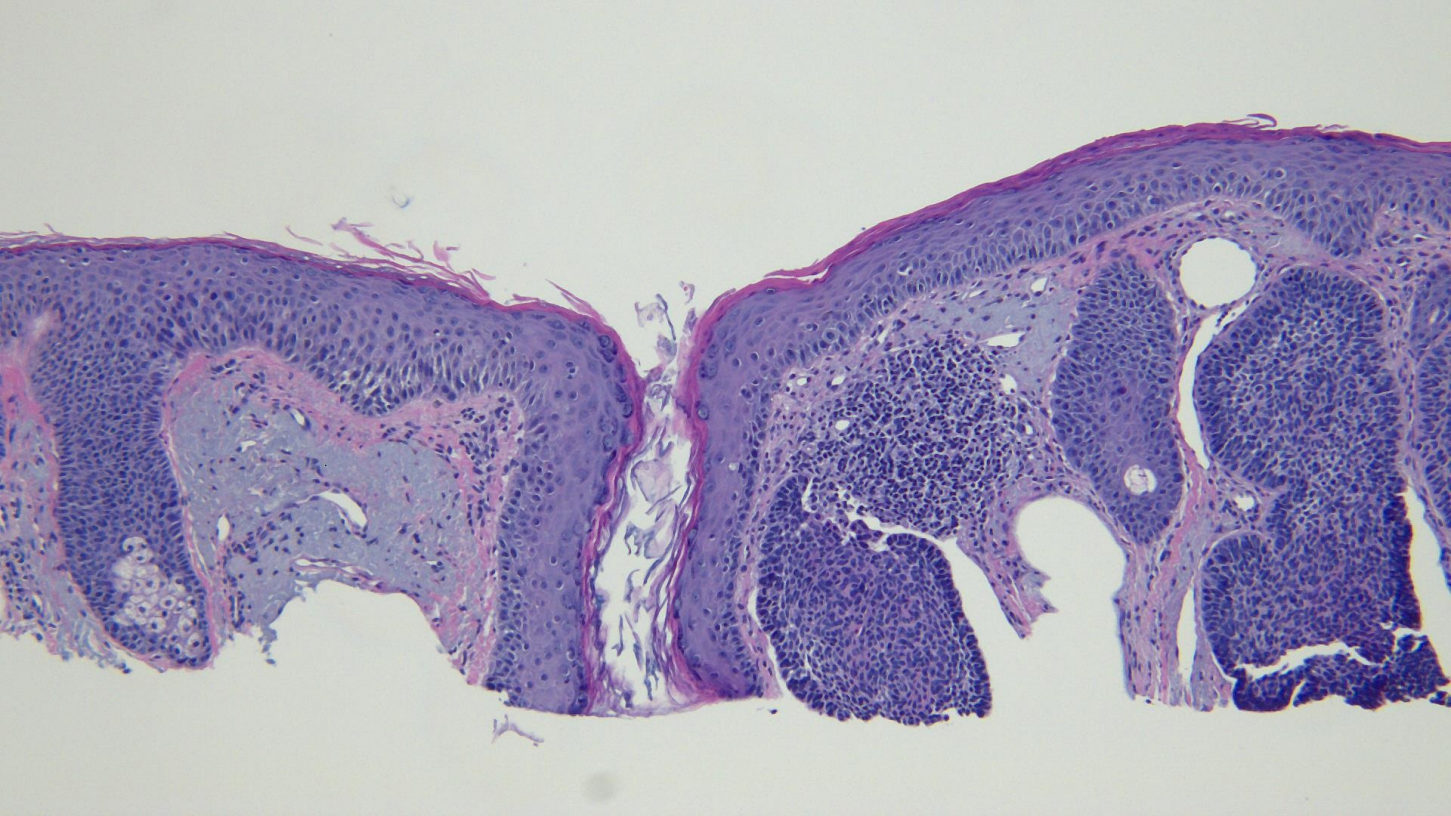
Microscopic examination of a red dot basal cell carcinoma on the left nostril of a 71-year-old male Intermediate magnification view of the biopsy of the red dot basal cell carcinoma from the left nostril shows extensive solar elastosis; in addition, telangiectasias are present in the papillary dermis adjacent to the tumor (hematoxylin and eosin, x 10).

**Figure 13:**
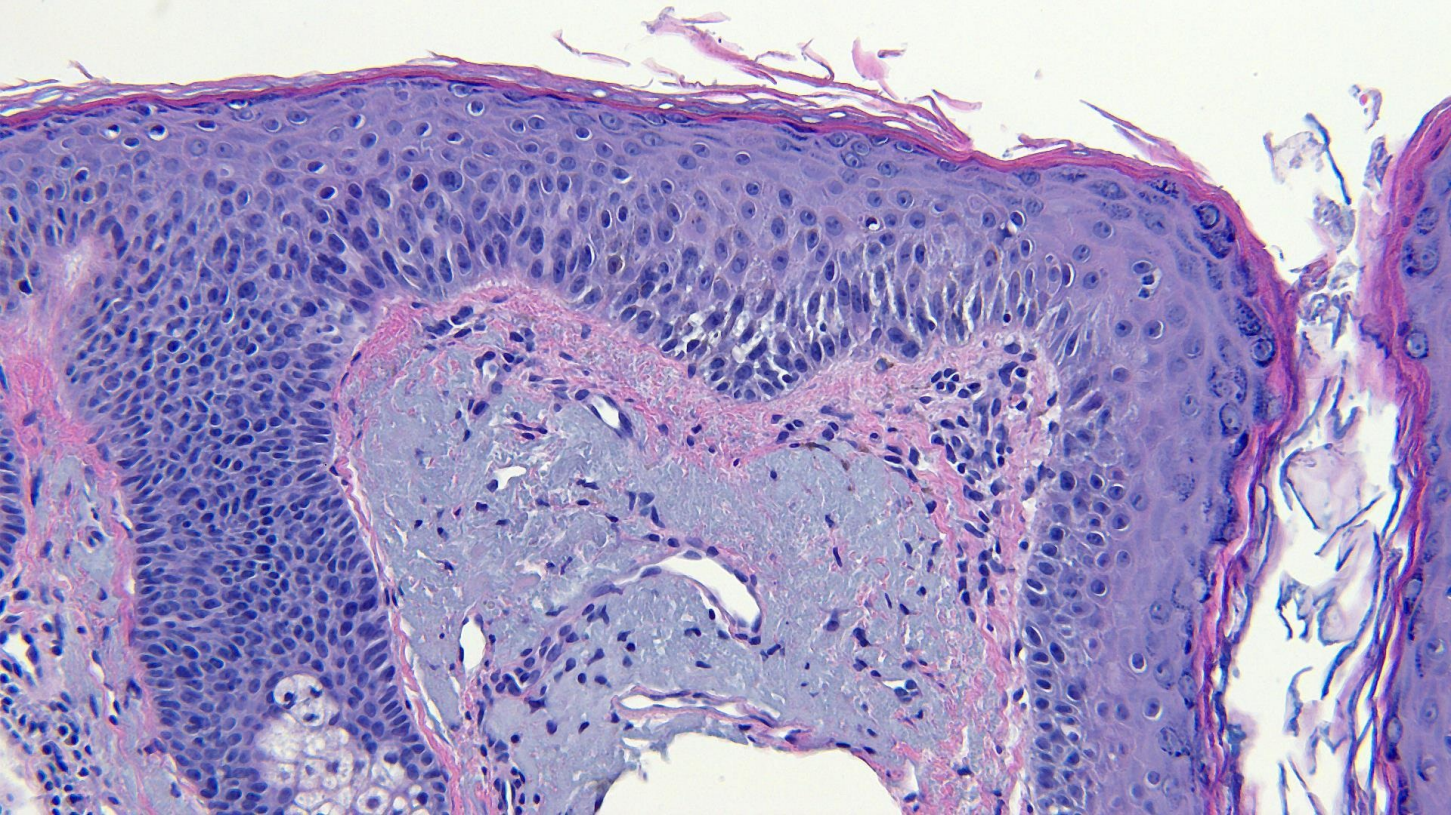
Microscopic examination of a red dot basal cell carcinoma on the left nostril of a 71-year-old male High magnification view of the biopsy of the red dot basal cell carcinoma from the left nostril shows dilated vessels that are present in the large areas of solar elastosis (hematoxylin and eosin, x 20).

**Figure 14:**
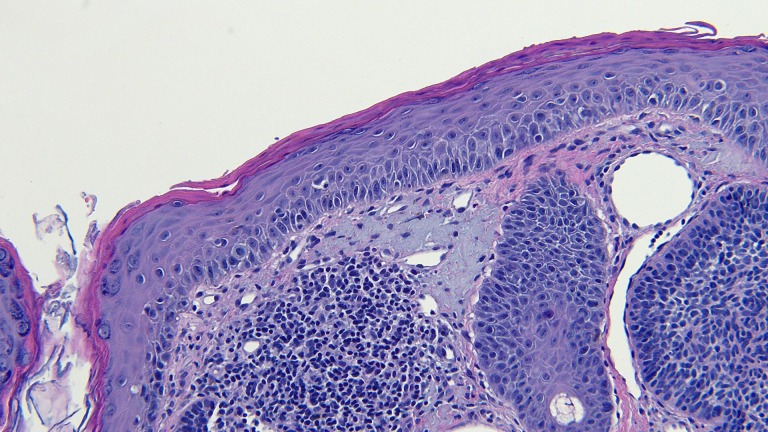
Microscopic examination of a red dot basal cell carcinoma on the left nostril of a 71-year-old male High magnification view of the biopsy of the red dot basal cell carcinoma from the left nostril shows that, in addition to tumor aggregates, hair follicles and perifollicular lymphocytic inflammation is also present (hematoxylin and eosin, x 20).

The actinic keratoses were treated with liquid nitrogen cryotherapy. The lesion on his left nostril was biopsied using the shave technique. Microscopic examination of the lesion showed nodular aggregates of basaloid tumor cells extended from the adjacent epidermis into the dermis. There were also telangiectatic blood vessels in the papillary dermis (Figures 11-14).

Correlation of the clinical presentation and pathologic findings of the nasal tip lesion was a red dot basal cell carcinoma (with nodular tumor aggregates). Excision of the tumor using the Mohs technique is planned.

### Case 3

A 74-year-old female presented for a complete skin examination in May 2016. She had a history of actinic keratoses and five basal cell carcinomas. There were several new skin lesions since her last visit six months earlier.

She also had a previous mantle cell lymphoma. In February 2015, she had achieved a clinical remission after receiving three cycles of Rituxan and Treanda that was followed by an autologous stem cell transplant in April 2015. She also has a past medical history of diabetes mellitus (controlled with diet), obstructive sleep apnea (treated with continuous positive airway pressure therapy) and a recent deep vein thrombosis (that is currently being treated with Coumadin).

**Figure 15:**
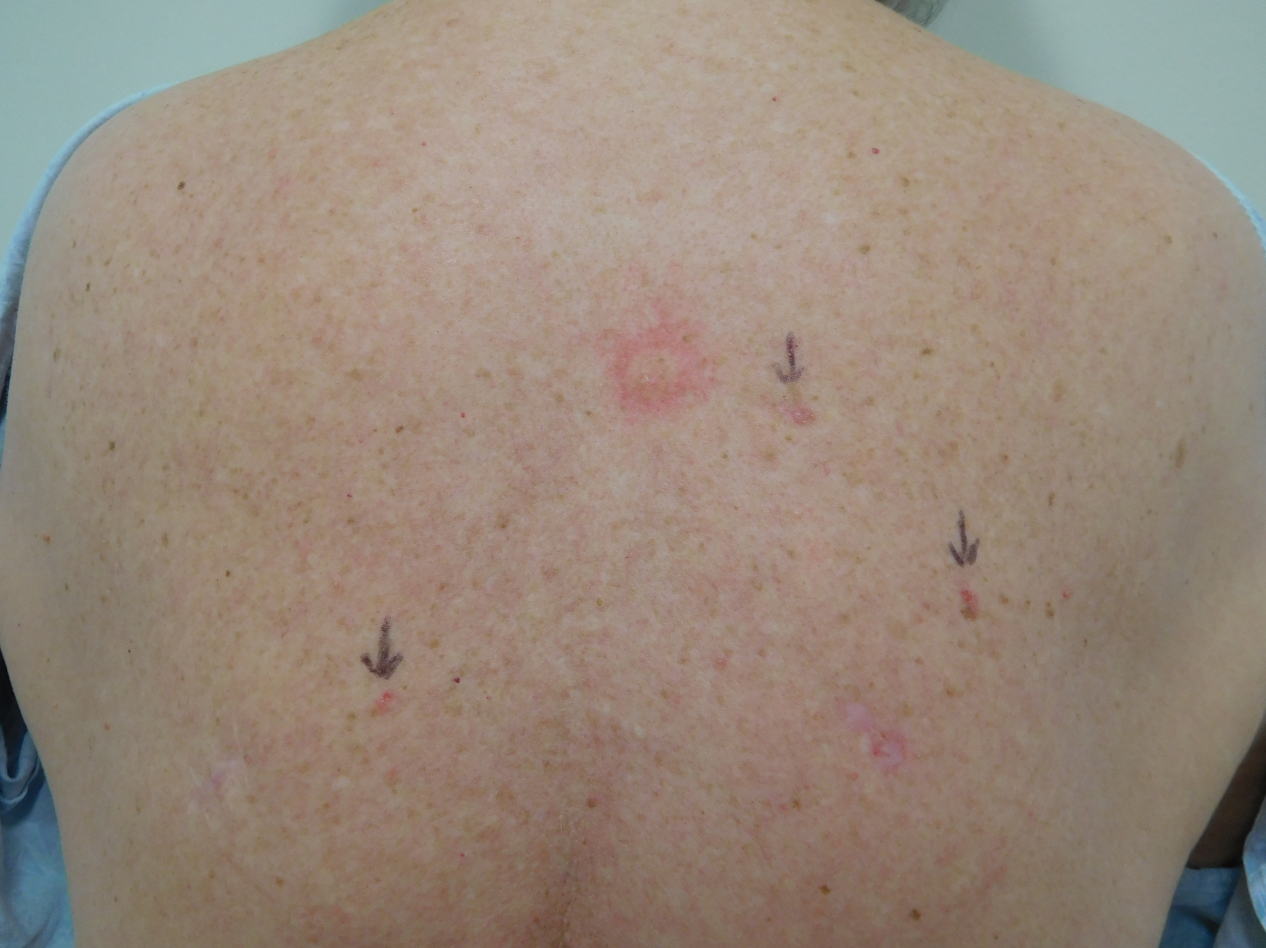
Clinical presentation of a red dot basal cell carcinoma on the left mid back of a 74-year-old female The upper back of a 74-year-old female shows an actinic keratosis that has been treated with liquid nitrogen cryotherapy in the upper central area. There are two red plaques (arrows) on her right upper central back and the right upper mid back; these are biopsy-confirmed superficial and nodular basal cell carcinomas. In addition, there is a 2 x 2 mm red dot within a 7 x 9 mm area of erythema on her left mid back (arrow) which is a red dot basal cell carcinoma with superficial and nodular pattern of tumor.

**Figure 16:**
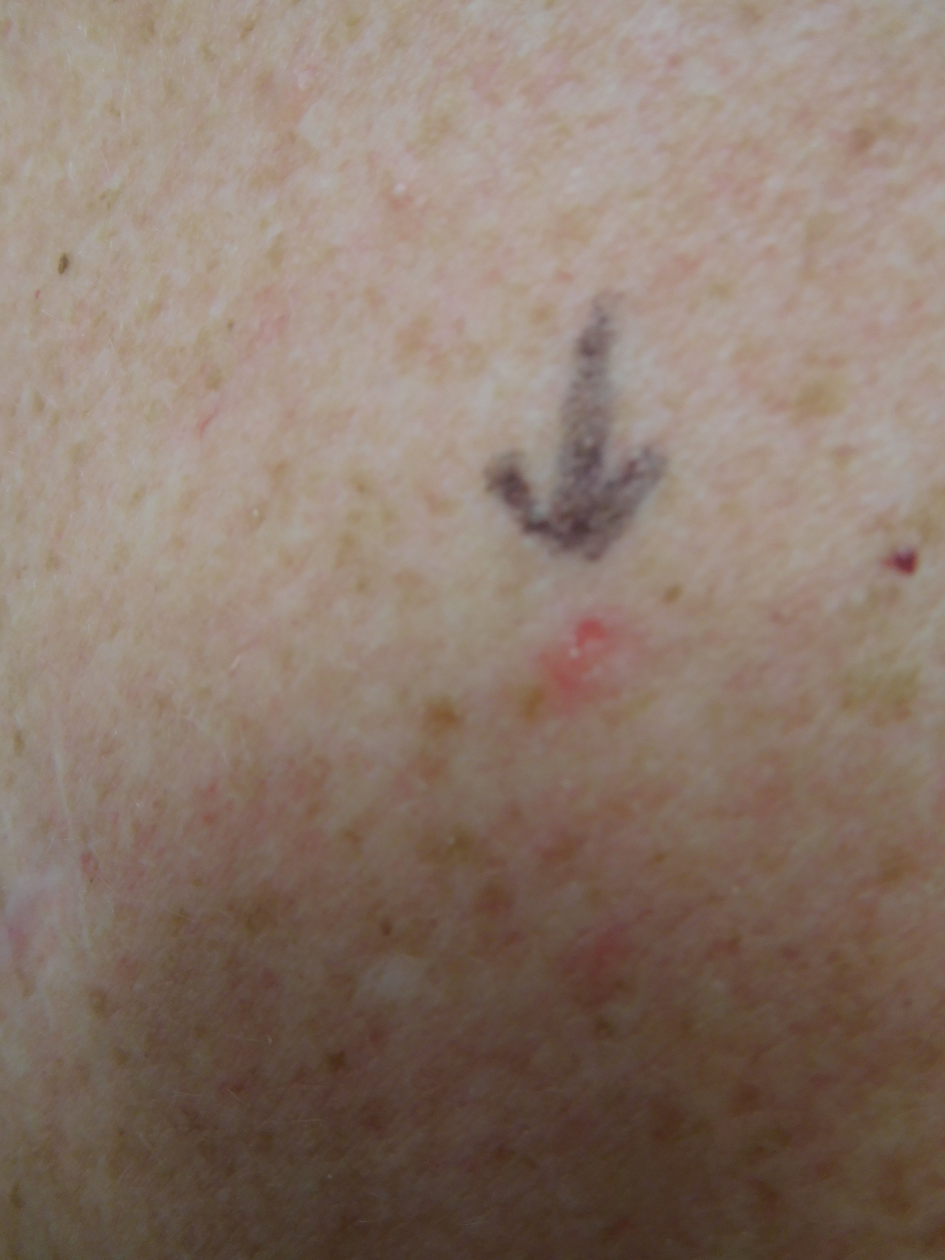
Clinical presentation of a red dot basal cell carcinoma on the left mid back of a 74-year-old female A closer view of the red dot basal cell carcinoma on the left mid back of a 74-year-old female.

**Figure 17:**
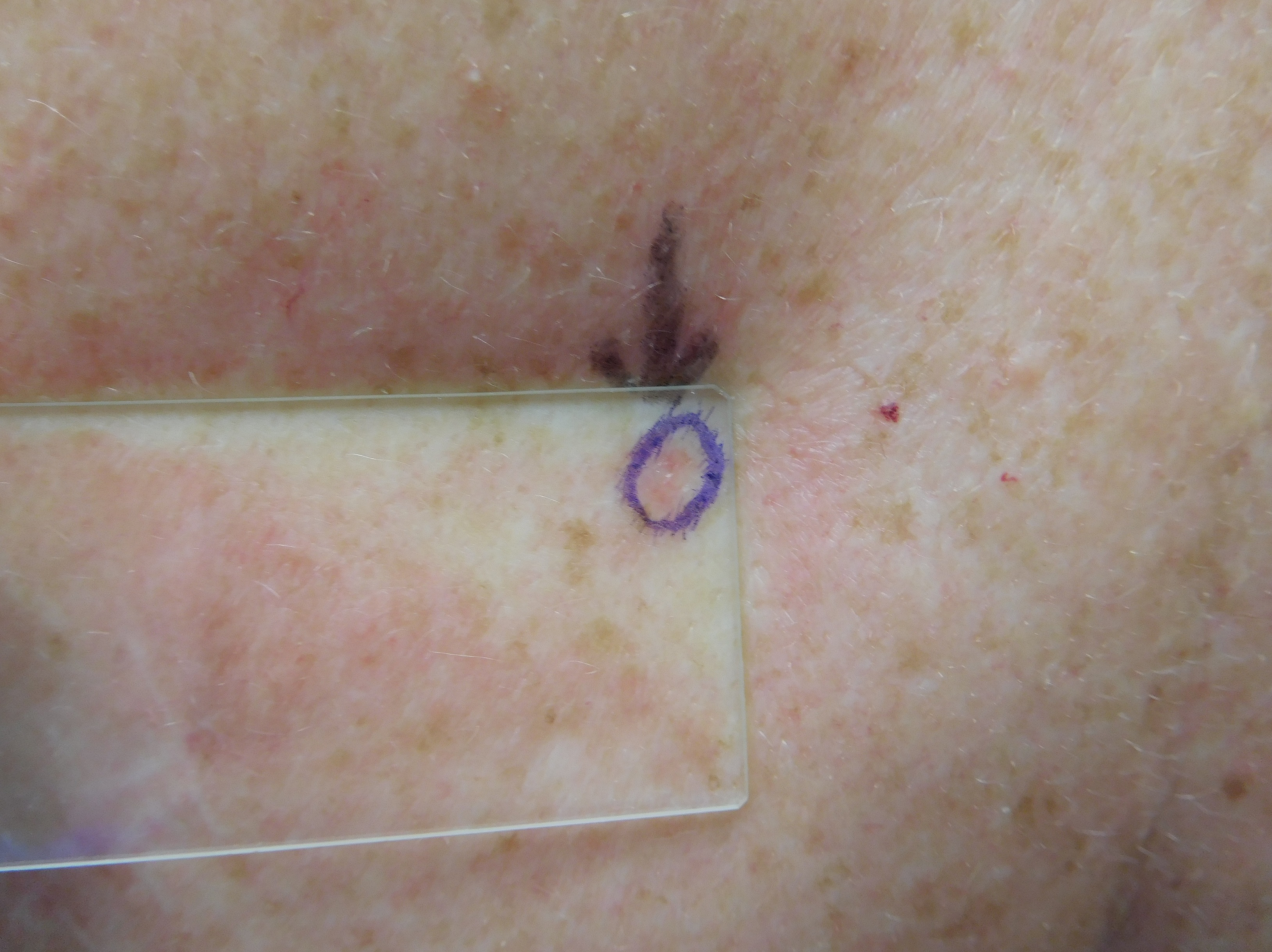
Diascopy of a red dot basal cell carcinoma on the left mid back of a 74-year-old female The red dot basal cell carcinoma on the left mid back is circled; the tumor blanches when a glass microscope slide is pressed against it.

The cutaneous examination was remarkable for four erythematous scaling plaques on her arms and back, consistent clinically with actinic keratoses. She had three red plaques, each approximately 6 x 4 mm, on her right upper central back, right upper mid back and right hip (Figure 15). In addition, she had a 2 x 2 mm red dot within a 7 x 9 mm area of erythema on her left mid back (Figures 15-16); the lesion blanched when pressed with a glass microscope slide (Figure 17).

**Figure 18:**
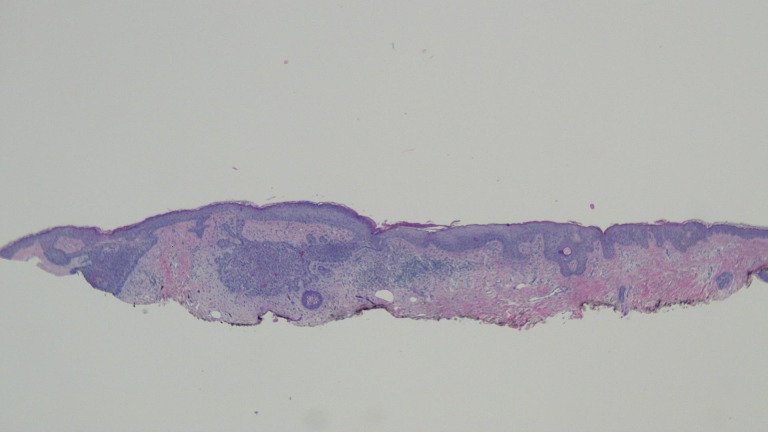
Microscopic examination of a red dot basal cell carcinoma on the left mid back of a 74-year-old female Low magnification view of the biopsy of the red dot basal cell carcinoma from the left mid back shows nodular aggregates and some superficial buds of basaloid tumor cells extending from the overlying epidermis into the underlying dermis (hematoxylin and eosin, x 2).

**Figure 19:**
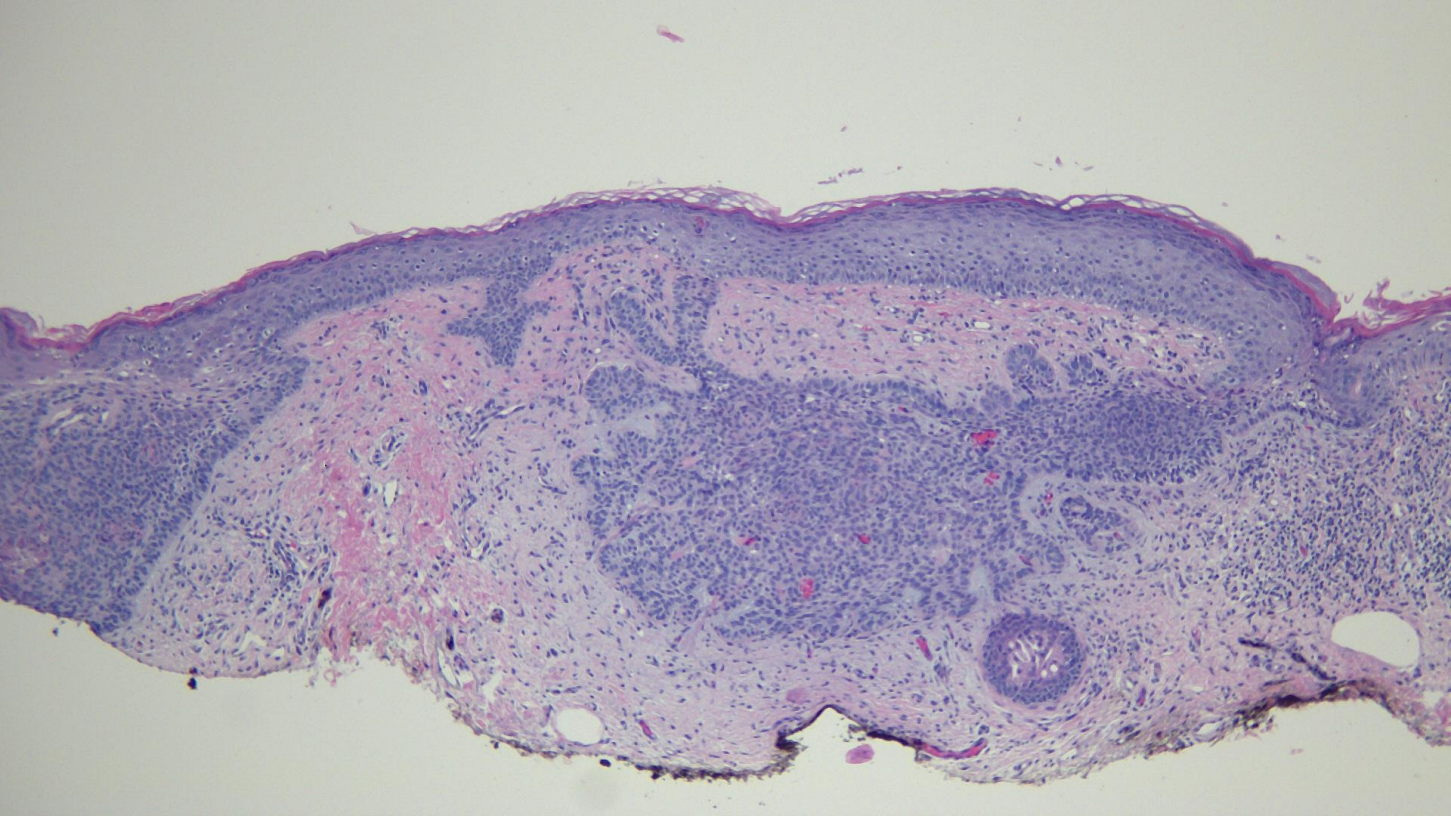
Microscopic examination of a red dot basal cell carcinoma on the left mid back of a 74-year-old female Intermediate magnification view of the biopsy of the red dot basal cell carcinoma from the left mid back shows vessels containing red blood cells within the tumor aggregates (hematoxylin and eosin, x 10).

**Figure 20:**
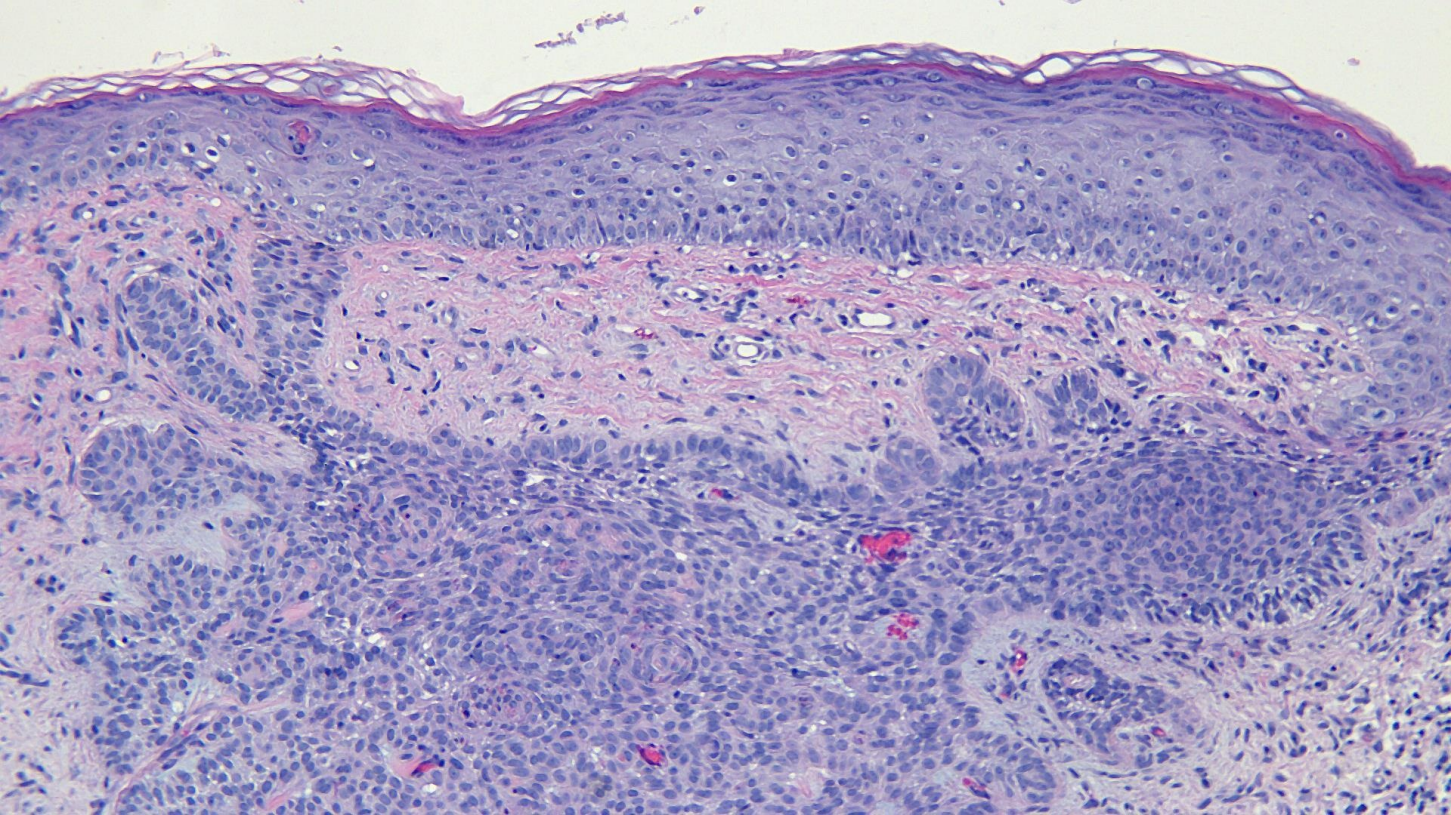
Microscopic examination of a red dot basal cell carcinoma on the left mid back of a 74-year-old female Intermediate magnification view of the biopsy of the red dot basal cell carcinoma from the left mid back shows several telangiectatic blood vessels in the stroma surrounding the tumor (hematoxylin and eosin, x 20).

**Figure 21:**
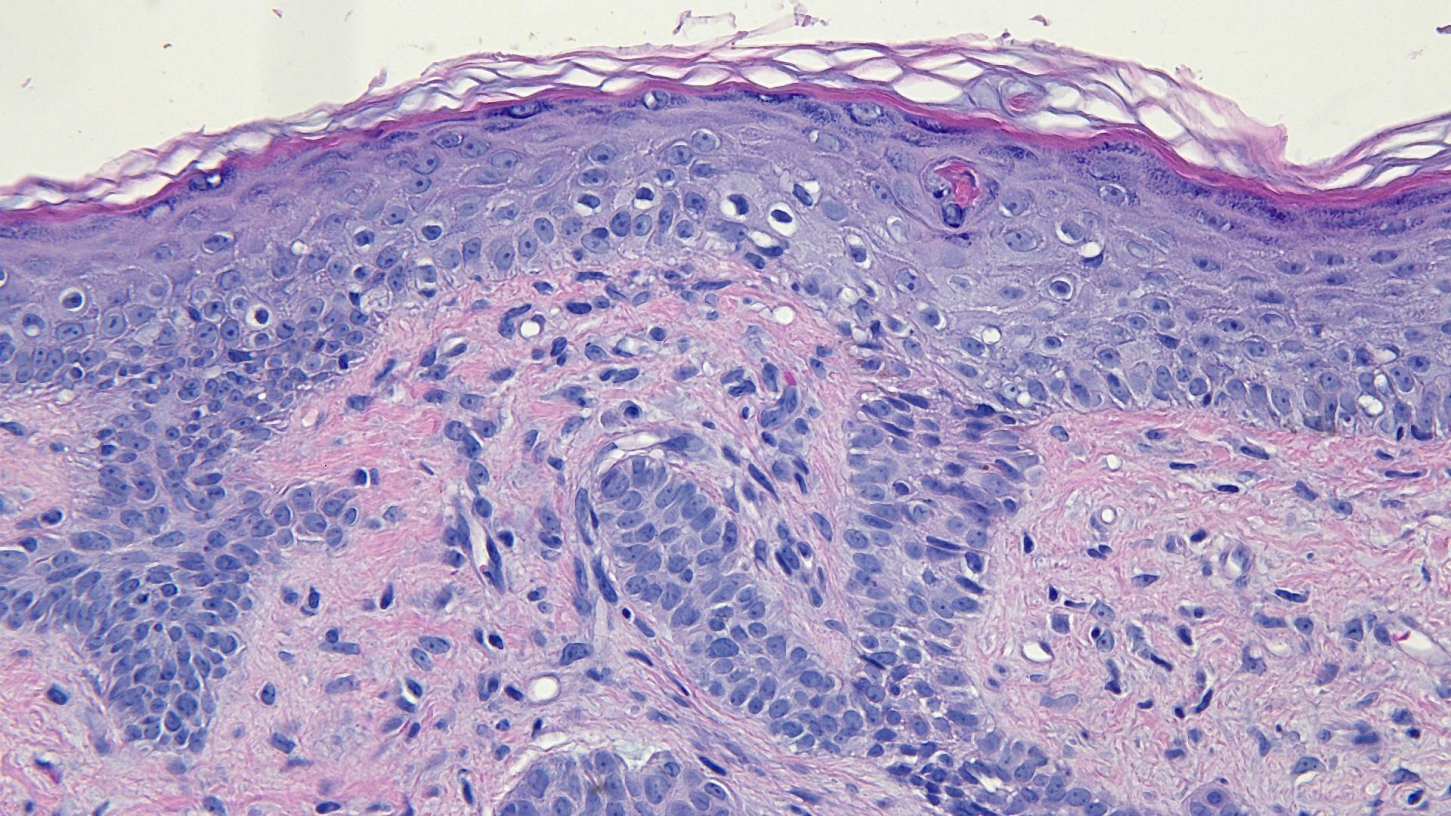
Microscopic examination of a red dot basal cell carcinoma on the left mid back of a 74-year-old female High magnification view of the biopsy of the red dot basal cell carcinoma from the left mid back shows numerous small blood vessels in the stroma surrounding the tumor (hematoxylin and eosin, x 40).

The actinic keratoses were treated with liquid nitrogen cryotherapy. The back lesions were each biopsied using a shave technique. The red dot on the left mid back showed superficial buds and nodular aggregates of basaloid tumor cells extending from the epidermis into the dermis; there were also telangiectatic blood vessels in the papillary dermis (Figures 18-21). The other back lesions and right hip lesion also showed similar appearing tumor cells along the basal layer of the epidermis and in the superficial dermis.

Correlation of the clinical presentation and pathologic findings of the left mid back lesion was a red dot basal cell carcinoma (with superficial and nodular tumor aggregates). The tumor was excised using the Mohs technique. Only one stage was required for cancer removal; the size of the defect was 15 x 11 mm. A complex layered closure was used to repair the surgical wound.

The three superficial and nodular basal cell carcinomas on the right back and right hip were also subsequently excised using the Mohs technique; the surgical wounds were closed with complex layered closures. Follow-up examination six months later showed excellent healing of the four surgical sites and no evidence of recurrence.

## Discussion

Basal cell carcinoma is the most common skin cancer [5-7]. Several clinical variants of basal cell carcinoma have been described. Albeit, uncommonly reported basal cell carcinoma can also present as a small red dot (red dot basal cell carcinoma) [1, 4].

Johnson, et al. reviewed unusual basal cell carcinomas in a paper published in Cutis in August 1994 [1]. In the section discussing morphologic characteristics, they commented that “unusual variants included the red dot basal cell carcinoma [1].” To the best of my knowledge, this is the first reference to this morphologic subtype of basal cell carcinoma.

Nearly two decades later, in October 2012, Tromberg, et al. commented that the red dot basal cell carcinoma is an early and distinct presentation of basal cell carcinoma [2]. In December 2014, Cohen reported a woman with red dot basal cell carcinoma [3]. Two years later, in May 2016, Loh and Cohen described another woman with red dot basal cell carcinoma [4].

Including this report, there are clinical images, reports of patient characteristics or both provided for seven individuals with red dot basal cell carcinoma (Table 1) [2, 4]; however, only limited information is provided in the figure legend for two of the patients [2]. There are two males and three females; the gender was not described for two of the patients. The patients ranged in age from 50 years to 74 years; the median age was 71 years. All of the patients were Caucasian.

**Table 1:** Characteristics of patients with red dot basal cell carcinoma [a,b] [a]Abbreviations: A: age (in years); AK: actinic keratosis; Arrhy: arrhythmia; BCC: basal cell carcinoma; Bl: blanchability; BPPV: benign paroxysmal positional vertigo; C: case; Ca: Caucasian; CAD: coronary artery disease; CLC: complex layered closure; CR: current report; DM: diabetes mellitus; DVT: deep vein thrombosis; ED&C: electrodessication and curettage; FTSG: full thickness skin graft; G: gender; HL: hyperlipidemia; HTN: hypertension; Hx: history of; L: left; Lo: location of basal cell carcinoma; M: male; MB: mid back; MCL: mantle cell lymphoma; MM: malignant melanoma (superficial spreading); Mohs: Mohs surgical technique for excision; NB: nasal bridge; Neph: nephrolithiasis; No: nostril; Nod: nodular; NS: not stated; NT: nasal tip; OAUB: overactive active urinary bladder; OSA: obstructive sleep apnea; Path: pathology subtype of basal cell carcinoma; PMHx: past medical history; PW: postoperative wound size in millimeters; R: recurrence of basal cell carcinoma; Ra: race; Rat: ratio of postoperative wound area to preoperative tumor area; Ref: reference; S1: one Mohs stage; S2: two Mohs stages; SCC: squamous cell carcinoma; Si: size (in millimeters of preoperative cancer); Sup: superficial; Th: thigh; Tx: treatment; W: woman; x: by; +: present; -: absent; &: and [b]Cases 6 and 7: Clinical photographs before and after Mohs surgery of two Caucasian patients with tumors on the left ala (preoperative size = 2 x 2 mm; postoperative wound = 4 x 5 mm; 5:1 ratio of postoperative wound area to preoperative tumor area) and on the right nasal sidewall (preoperative size = 4 x 4 mm; postoperative wound = 15 x 15 mm; 14:1 ratio of postoperative wound area to preoperative tumor area) [2].

C	A Ra G	Lo	Si	Bl	Path	Tx	PW	Rat	R	Hx AK	Hx Skin Cancer	PMHx	Ref
1	70 Ca M	NT	3x3	NS	Sup & Nod	Mohs S2 FTSG	11 x 10	12 : 1	-	+	4BCC	BPPV CAD Gout HL Neph	CR C1
2	71 Ca M	L No	2x2	-	Nod	Mohs	NS	NS	-	+	1BCC 1SCC	CAD HL	CR C2
3	50 Ca W	L Th	3x3	NS	Nod	ED & C	NS	NS	-	+	4BCC 1SCC 2MM	NS	3
4	72 Ca W	L NB	2x3	+	Sup	Mohs S2 FTSG	10 x 10	17 : 1	-	+	2BCC 1SCC	Arrhy HTN OAUB	4
5	74 Ca W	L MB	7x9	+	Sup & Nod	Mohs S1 CLC	15 x 11	3 : 1	-	+	8BCC	DM DVT MCL OSA	CR C3

All of the patients had a history of actinic keratoses and non-melanoma skin cancers. Including the visit when the patients had their red dot basal cell carcinoma diagnosed, the individuals had between one to eight additional basal cell carcinomas (median, four basal cell carcinomas). Two of the females also had a squamous cell carcinoma. The 50-year-old female had a prior tumor whose morphology was similar to her current red dot basal cell carcinoma; she also had a history of two prior primary superficial spreading malignant melanomas [3].

Johnson, et al. commented that red dot basal cell carcinomas usually occurred on sun-exposed sites [1]. Indeed, the most common location for red dot basal cell carcinoma was the nose (five of seven patients, 71%). One of these patients, the 70-year-old male, also had a concurrent pigmented basal cell carcinoma on his back. The other sites of red dot basal cell carcinoma included the back and the thigh, each in one patient. Both of these individuals had concurrent non-melanoma skin cancers: the 50-year-old female had two additional basal cell carcinomas on her left arm and left thigh and a squamous cell carcinoma on her left arm [3], and the 74-year-old female had three additional basal cell carcinomas on her right back (two cancers) and right hip (one cancer).

The size of the red dot basal cell carcinomas ranged from 2 x 2 mm to 7 x 9 mm; the median size was 3 x 3 mm. The cancer often presented as a solitary small red macule or papule. However, in some of the patients, the tumor appeared as a red area surrounded by either erythema or a flesh-colored papule; individuals with the latter appearance of a skin-colored halo surrounding the red dot, such as the nasal tip tumor in the 70-year-old male, prompted Johnson, et al. to also refer this presentation of red dot basal cell carcinomas as halo basal cell carcinoma [1].

Tromberg, et al. also noted a halo appearance to the red dot basal cell carcinoma [2]. However, they were able to create the halo after manipulating the skin adjacent to the tumor. Specifically, they observed that a subtle shiny induration or pearling of the epidermis around the red dot could be seen when the surrounding skin was stretched [2]. 

Morphologically, red dot basal cell carcinoma mimics benign vascular lesions, such as a hemangioma or a telangiectasia. Indeed, in the 72-year-old female with the left nasal bridge carcinoma, the clinical impression of a telangiectasia resulted in the biopsy of the lesion being postponed for three months [4]. Therefore, early diagnosis of this variant of basal cell carcinoma requires a high index of suspicion [1]. However, the appearance of a new red dot on a sun-exposed site in a patient with a personal history of actinic keratoses or non-melanoma skin cancer or both may prompt the clinician to consider performing additional evaluations of the skin lesion.

Evaluation of a skin lesion, by pressing a glass microscope slide against it, can be performed to assess for blanchability. This test, referred to as diascopy, involves using a clear material to depress a lesion in order to observe blood dissipating intravascularly and the tissue acquiring a blanched appearance [8]. Vascular lesions, such as telangiectasia, blanch after diascopy and skin cancers, such as basal cell carcinomas typically persist without blanching [4].

The red dot basal cell carcinoma on the 71-year-old male's left nostril did not blanch, prompting a biopsy to be performed. However, both of the red dot basal cell carcinomas on the left nasal bridge tumor of the 72-year-old female [4] and the left mid back of the 74-year-old female blanched after diascopy. Therefore, persistence of the red dot after pressing against it with a glass microscope slide, as demonstrated by the left nostril tumor of the 71-year-old male, supports the suspected diagnosis of basal cell carcinoma and should prompt additional evaluation, such as a skin biopsy, to confirm the diagnosis. However, blanching of a red dot skin lesion during diascopy, when the possibility of skin cancer is being entertained, cannot be relied upon to definitively differentiate between red dot basal cell carcinoma (which can contain dilated blood vessels) and telangiectasia; hence, under these circumstances, dermoscopy or biopsy or both should be considered.

Dermoscopy is a noninvasive technique that permits in vivo observation, with magnification, of the structures in the epidermis and papillary dermis. It is a valuable tool for evaluation of pigmented lesions. It is also useful for examination of nonpigmented skin disorders such as skin cancers, inflammatory conditions and infectious diseases [7, 9-10].

Hemangiomas are benign vascular lesions characterized by the proliferation, with or without dilatation of capillaries. Dermoscopically, they are characterized by well-demarcated red, maroon, blue or black lacunae. Telangiectasia dilated blood vessels—of varying diameter—in the superficial dermis, can be an incidental finding in sun-exposed skin of elderly individuals [9]. However, telangiectasias are frequently a component of basal cell carcinomas [7, 10].

Dermoscopically arborizing vessels and short fine telangiectasias are the most common vascular patterns in nodular basal cell carcinoma and superficial basal cell carcinoma, respectively. In addition, yet less frequently, other vascular patterns (such as hair pin, glomerular, dotted, comma and polymorphous vessels), ulceration, blue-gray globules, blue-gray ovoid nests, leaf-like areas, spoke wheel areas and white shiny structures can also be present in non-pigmented and pigmented basal cell carcinomas [7, 10]. Hence, the presence of abnormal vascular patterns, with or without other dermoscopic changes, may permit differentiation between red dot basal cell carcinoma and hemangioma or telangiectasia; however, to date, red dot basal cell carcinoma has not been evaluated with dermoscopy.

Microscopic examination of the red dot basal cell carcinomas observed by Cohen [3], Loh and Cohen [4], and in the patients described in this report all demonstrated histologically non-aggressive pathology subtypes of basal cell carcinoma: either superficial pattern (one patient), nodular pattern (two patients) or both superficial and nodular patterns (two patients). In addition, in the papillary dermis, there are not only numerous endothelial-lined blood vessels but also extravasated erythrocytes; it is postulated that these microscopic findings correlate with the appearance of the red dot that is observed clinically. Also, the partial or complete blanching observed during diascopy that was observed in two of the patients’ tumors might have resulted from compression of these abundant vessels in the dermis adjacent to the carcinoma.

However, Tromberg, et al. did not observe any correlation between the clinical presentation of red dot basal cell carcinoma and respective histologic pattern of the tumor in their patients [2]. Similar to the patients in this and other [3-4] reports, some of their patients also had tumors demonstrating less aggressive nodular and superficial histology [2]. Yet, the skin cancer was unexpectedly characterized by an aggressive subtype of pathology—with an extensive pattern of invasive strands of basal cells—in other individuals with red dot basal cell carcinoma [2].

The treatment of choice for red dot basal cell carcinoma is excision using the Mohs surgical technique. This modality was performed in six of seven (86%) of the patients. The number of stages required to remove the tumor was described in three of the individuals [four current reports]; the cancer was cleared after one (one woman) or two (two patients) stages. The postoperative wounds were repaired with either a full thickness skin graft (on the nose of two patients) or a complex layered closure (on the back of one female). Electrodesiccation and curettage of the red dot basal cell carcinoma on the left thigh were performed in the 50-year-old female who refused to have her cancer excised [3].

Tromberg, et al. commented that the red dot basal cell carcinomas were frequently early cancers [2]. They noted that, despite their indistinct clinical margins, the wounds following removal of the tumors were frequently small. Indeed, a comparison of the postoperative wound area to the preoperative cancer area was 5:1 in their patient with a left ala carcinoma [2]. Similarly, the ratio of the postoperative wound to preoperative carcinoma area was only 3:1 in the 74-year-old female with a red dot basal cell carcinoma on her left mid back.

However, for several patients with red dot basal cell carcinoma on the nose, the lateral spread of the tumor was more significant than the clinical margins of the cancer would have suggested. Indeed, for three of the patients, the ratio of the postoperative wound to preoperative carcinoma area ranged from 12:1 (in the 70-year-old male with a nasal tip cancer) to 17:1 (in the 72-year-old female with a left nasal bridge cancer); the median was 14:1 in the patient of Tromberg, et al. with the right nasal sidewall cancer) [2].

Follow-up after surgery is limited. It only ranged from four to six months in five of the patients. However, to date, there has been no recurrence of the red dot basal cell carcinomas (Table 1) [2, 4], [current report].

## Conclusions

Red dot basal cell carcinoma, a distinct variant of basal cell carcinoma that clinically can mimic a hemangioma or telangiectasia, has been reported in seven Caucasian patients (median age of 71 years at diagnosis) with a history of either actinic keratosis, non-melanoma skin cancer and/or melanoma. The nose was the most common site. The tumors had a median size of 3 x 3 mm and appeared as a solitary red macule or papule with or without surrounding erythema or a flesh-colored papule. Diascopy, which typically results in blanching of a vascular lesion, can be misleading in the clinical evaluation of a suspected red dot basal cell carcinoma since some of the patients’ tumors blanched after a glass slide was pressed against them. Dermoscopy may be helpful in differentiating red dot basal cell carcinoma from benign vascular lesions. The pathology of red dot basal cell carcinoma varied from a less aggressive histology (nodular or superficial or both) to an aggressive pattern of invasive tumor cell strands. The treatment of choice for red dot basal cell carcinoma is excision using the Mohs surgical technique. There has been no recurrence following treatment. In conclusion, the appearance of a new red dot on a sun-exposed site in a patient with a personal history of actinic keratoses or non-melanoma skin cancer should prompt the clinician to not only consider the possibility of red dot basal cell carcinoma but also to perform an additional evaluation of the skin lesion.
